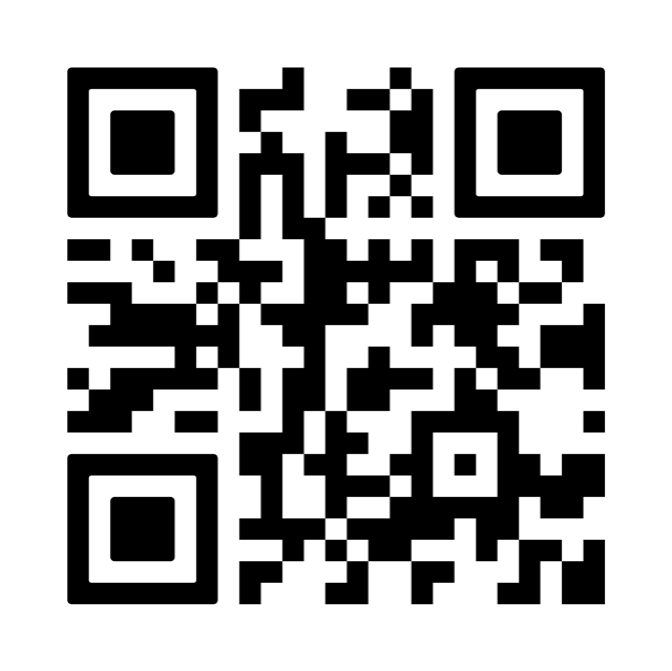# Pelvic anatomy in laparoscopic surgery for pelvic organ prolapse: dissect your success

**DOI:** 10.52054/FVVO.14.4.047

**Published:** 2023-01-27

**Authors:** M.G. Melo, R Botchorishvili

**Affiliations:** Department of Gynaecological Surgery, Centre Hospitalier Universitaire (CHU) Estaing de Clermont-Ferrand, 63100 Clermont-Ferrand, France; Centre International de Chirurgie Endoscopique (CICE), 63000 Clermont-Ferrand, France; Department of Gynaecology and Obstetrics, Hospital Vila Franca de Xira (HVFX), 2600-009 Vila Franca de Xira, Portugal

**Keywords:** Laparoscopy, pelvic organ prolapse, colposacropexy, anatomy, dissection

## Abstract

**Background:**

Laparoscopic surgery for pelvic organ prolapse is a complex procedure, requiring high technical skills and great knowledge of the anatomy to perform a safe dissection and achieve the best clinical and surgical outcomes.

**Objectives:**

To highlight the anatomical landmarks during dissection in this procedure and give tips for a safer and more effective performance.

**Materials and methods:**

Surgical videos of the dissection involved in laparoscopic surgery for pelvic organ prolapse in a stepwise approach.

**Main outcome measures:**

Identification of the most important anatomical landmarks involved in the dissection of the promontory, the para-rectal space, the recto-vaginal space, and the vesico-vaginal space. Advice for acquiring better exposure and the right cleavage planes. Presentation of some difficult cases during dissection.

**Results:**

Step-by-step overview of the different steps of dissection involved in laparoscopic surgery for pelvic organ prolapse, specifying the most important anatomical landmarks for reference and at risk of damage and presenting tips to correctly perform the dissection.

**Conclusion:**

Besides the great surgical technical skills required, deep knowledge of pelvic anatomy is key for performing laparoscopic surgery for pelvic organ prolapse safely, minimising complications and recurrence and improving quality of life and the overall success of surgery.

## Learning objective

As a complex surgical procedure, laparoscopic surgery for pelvic organ prolapse involves careful dissection and correct identification of the anatomical structures to prevent complications and recurrence and achieve better clinical and surgical outcomes. This video article aims to highlight the anatomical landmarks involved in the dissection during laparoscopic surgery for pelvic organ prolapse and give advice for a safer and more effective dissection.

## Introduction

Laparoscopic surgery for pelvic organ prolapse is a complex surgical procedure, requiring advanced technical skills. The surgical dissection involved in this procedure, namely the dissection of the promontory, involves risk of damage of important structures in proximity, including vascular structures, nerves, and the right ureter. Therefore, great knowledge of the pelvic anatomy is mandatory to perform the dissection with safety, prevent immediate and late complications, minimise prolapse-related symptoms, and improve quality of life (Giraudet et al., 2018; Serbetcioglu et al., 2020). In addition, correct dissection for later placement of the mesh, with preservation of important structures such as the nerves, highly contributes to anatomic success of the surgery (Serbetcioglu et al., 2020).

## Patients and methods

We present a video to set forth the different steps of the surgical dissection involved in laparoscopic surgery for pelvic organ prolapse in a stepwise approach, namely the dissection of the promontory, the para-rectal space, the recto-vaginal space, and the vesico-vaginal space (Ercoli et al., 2017). We selected a set of surgical videos to illustrate and highlight the anatomical structures that can be encountered during these steps, present some cases of higher complexity during dissection due to anatomical variation and offer advice for acquiring better exposure and the correct cleavage planes. No patient identifiable data were used.

## Results

A step-by-step overview of the surgical dissection involved in laparoscopic surgery for pelvic organ prolapse was performed.

First, we started by presenting the dissection of the promontory, which aims to reach the anterior longitudinal ligament to attach the mesh later in the procedure. We pointed out the safer area to perform this dissection, in a triangular region in proximity to the aortic bifurcation, the left common iliac vein, the middle sacral vessels, the right ureter and the right hypogastric nerve (Cosma et al., 2013; Giraudet et al., 2018; Muavha et al., 2019).

Secondly, we presented the dissection of the para-rectal space and underlined the potential anatomical structures at risk of damage in the pelvic side wall, namely the uterine artery, the ureter, the internal iliac vein, and the right inferior hypogastric nerve.

We proceeded to dissect the recto-vaginal space, drawing attention to the vagina, the rectum, the recto-vaginal ligament, the middle rectal artery and the puborectalis portion of the levator ani muscles.

Finally, we presented the dissection of the vesico- vaginal space, noting the vagina, the bladder, the vesico-uterine ligaments, and the ureters.

Throughout the video, the different anatomical structures in each step of the dissection are highlighted. Advice is given on how to optimise exposure to perform the dissection, by using tissue retraction systems. Dissection techniques and choosing the correct plane, utilising the champagne effect is also discussed.

## Discussion

Laparoscopic surgery for pelvic organ prolapse, which has been performed since 1992, is one of the preferred routes of surgical treatment of prolapse, especially apical, due to being a minimally invasive and effective technique (Ercoli et al., 2016; Serbetcioglu et al., 2020).

The dissection includes a risk of potential damage to important structures, which can be minimised by appreciating the pelvic anatomy and correctly identifying the anatomical structures in each step of surgery. By avoiding lesion of the vascular structures, haemorrhage is prevented, which could be potentially life-threatening (Giraudet et al., 2018). Additionally, by sparing the nerves, voiding, bowel, and sexual dysfunction is minimised, improving quality of life (Giraudet et al., 2018; Serbetcioglu et al., 2020). Post laparoscopic sacropexy obstructed defecation syndrome was described in 22%, in a series of 18 patients. In that study, a statistically significant association between bowel dysfunction and medial midline dissection of the promontory was observed, where the distal portion of the superior hypogastric plexus and the right hypogastric nerve lie (Cosma et al., 2013). The safer triangular area of dissection of the promontory was described to avoid neural and vascular damage. Dissection should be performed medially and close to the right common iliac artery to preserve the nerves, which run on the left of the midline (Cosma et al., 2017; Giraudet et al., 2018). A later study comparing a nerve-sparing technique vs the standard technique showed no cases of obstructive defecation syndrome (0/25) vs 4 in the standard group (4/18), with a p-value of 0.02 (Cosma et al., 2017).

In terms of long-term anatomical result and recurrence, in one uni-centric study including 115 patients with apical prolapse who underwent laparoscopic nerve-sparing sacrocolpopexy, no apical prolapse was found in the 10% of the patients that presented with recurrence and all of them were asymptomatic (Serbetcioglu et al., 2020).

To the best of our knowledge, this is the first video-article pointing out the anatomical landmarks involved in all steps of dissection of laparoscopic surgery for pelvic organ prolapse, presenting, as well, tips to consider for a careful and correct dissection.

## Conclusions

The performance of laparoscopic surgery for pelvic organ prolapse requires not only great surgical technical skills, but also a deep knowledge of the pelvic anatomy. The correct dissection of the pelvic spaces and anatomical structures involved in this procedure provides greater safety, by preserving relevant anatomical structures and avoiding intraoperative and postoperative complications. Additionally, a precise dissection is essential to achieve a satisfying anatomical result and minimise the risk of recurrence. Consequently, knowing the anatomical landmarks and the right dissection technique is key to success of surgery.

## Video scan (read QR)

https://vimeo.com/786591464/d9724dc8bd

**Figure:**